# Potential in-host evolution of *Klebsiella pneumoniae* ST147: convergence and the role of capsular alterations in morphotype diversity

**DOI:** 10.1128/spectrum.00170-25

**Published:** 2025-07-18

**Authors:** Katharina Sydow, Eyüp Doğan, Michael Schwabe, Stefan E. Heiden, Muhammad Moman Khan, Justus U. Müller, Jürgen A. Bohnert, Daniel Baecker, Rabea Schlüter, Peter Schierack, Elias Eger, Evgeny A. Idelevich, Karsten Becker, Katharina Schaufler

**Affiliations:** 1 Department of Epidemiology and Ecology of Antimicrobial Resistance, Helmholtz Institute for One Health, Helmholtz Center for Infection Research HZI, Greifswald, Germany; 2 Friedrich Loeffler-Institute of Medical Microbiology, University Medicine Greifswald 60634, Greifswald, Germany; 3 Faculty of Environment and Natural Sciences, Brandenburg University of Technology Cottbus-Senftenberg, Senftenberg, Germany; 4 Department of Pharmaceutical and Medicinal Chemistry, Freie Universität Berlin 9166, Berlin, Germany; 5 Imaging Center of the Department of Biology, University of Greifswald 26552, Greifswald, Germany; 6 Institute of Medical Microbiology, University Hospital Münster 39069, Münster, Germany; 7 University Medicine Greifswald 60634, Greifswald, Germany; McGill University, Ste-Anne-de-Bellevue, Quebec, Canada

**Keywords:** *K. pneumoniae*, in-host evolution, convergent pathotype, SCV, capsule deficiencies

## Abstract

*Klebsiella pneumoniae*, an important opportunistic pathogen, is traditionally classified into classic and hypervirulent pathotypes. Convergent strains combining antimicrobial resistance (AMR) and hypervirulence have emerged globally, posing a significant health challenge. In this study, we investigated potential in-host evolution and morphotypic variation among consecutive *K. pneumoniae* ST147 isolates from a single patient. As described in a previous study, the isolates displayed distinct morphotypes—small white, normal white, gray, and gray/dry (g/d) colonies. In this study, we now present new genomic and phenotypic analyses, which suggest the acquisition of AMR and virulence genes through plasmid gain. The early isolates carried fewer plasmids, resulting in low resistance and virulence, while later isolates acquired a hybrid IncFIB-IncHI1B plasmid encoding different resistance and aerobactin genes, significantly increasing their geno- and phenotypic AMR and virulence potential. Chromosomal integration of this plasmid in one isolate stabilized the acquired traits. Disruptions in the K locus, mediated by insertion sequences, were linked to the gray and g/d morphotypes, impairing capsule biosynthesis. Uronic acid assays confirmed reduced capsule production in these isolates. In contrast, small colony variants showed significant transcriptomic changes, including upregulation of capsule biosynthesis, iron uptake pathways, and AMR genes, suggesting persistence through altered metabolism. Our findings suggest in-host microevolution of *K. pneumoniae* ST147 from a classic to a convergent pathotype and highlight the genomic and transcriptomic adaptations underlying morphotypic diversity, providing new insights into the pathogen’s adaptability and persistence in certain environments.

**IMPORTANCE:**

*Klebsiella pneumoniae* is an important bacterial pathogen that can cause severe infections, particularly in healthcare settings. Traditionally, strains are classified as either drug-resistant or highly virulent, but recent strains combining both features have led to hard-to-treat infections. In this study, we investigated the potential in-host evolution of *K. pneumoniae* ST147 isolates from a single patient, revealing key genomic and phenotypic changes driving their adaptability. Over time, isolates acquired additional plasmids carrying antimicrobial resistance and virulence genes, including a hybrid plasmid integrated into the bacterial chromosome, stabilizing its beneficial traits. In addition, morphotypic diversity emerged, linked to genomic disruptions in capsule biosynthesis genes (K locus). While capsule-deficient morphotypes displayed structural changes, small colony variants exhibited transcriptomic adaptations to persist under stress. This work underscores the dynamic evolutionary capacity of *K. pneumoniae* adapting within the host, providing critical insights into its persistence, resilience, and the challenges posed by emerging convergent strains in clinical settings.

## INTRODUCTION

*Klebsiella pneumoniae*, a natural component of the human gut microbiome, is an opportunistic pathogen associated with various infections (1). Traditionally, *K. pneumoniae* is divided into two pathotypes: classic (cKp) and hypervirulent (hvKp) (1). While cKp is often multidrug resistant (MDR), it typically causes healthcare-associated infections such as pneumonia, urinary tract infections, and bacteremia in immunocompromised individuals (1). In contrast, hvKp strains are known to cause severe and invasive community-acquired infections, although hvKp is usually antibiotic susceptible (1). Alarmingly, a convergent pathotype combining hypervirulence with MDR has recently emerged (2–5).

The production of capsular polysaccharides, which are important for virulence, is a hallmark of all *K. pneumoniae*. However, hvKp strains usually produce these in greater quantities and often exhibit a hypermucoviscous (HMV) phenotype, associated with pathogenicity (6). Mutations in capsule biosynthesis genes can lead to excessive capsule formation or capsule deficiency (7), emphasizing their dynamic role in pathogenic evolution. Among genetic regulators of capsule formation, the *rmp* locus, mainly encoded on plasmids, regulates the HMV phenotype (6, 8, 9). Interestingly, plasmids encoding the *rmp* locus frequently combine genes for virulence and MDR (2). These mobile genetic elements can integrate into the chromosome, ensuring stable inheritance of beneficial traits (10).

We have previously reported *K. pneumoniae* isolates belonging to the international, high-risk sequence type (ST)147 with different colony morphologies on blood agar (11). These isolates were obtained during routine diagnostics at five different time points from urine and tissue samples from a patient with a shrapnel shell injury (11). Four distinct phenotypes were identified among the isolates from the last two time points: (i) white, (ii) gray with a smooth and shiny surface, (iii) gray and dry (g/d) colonies, and (iv) small colony variants (SCVs), which were white and smooth (11). When sub-cultivated on blood agar plates, the small colonies steadily split again into the four different morphotypes, a phenomenon that we subsequently termed “hyper-splitting.” Antibiotic susceptibility testing revealed variability in minimum inhibitory concentrations across isolates. Importantly, SCVs represent a particular adaptive strategy in bacterial pathogens, including *K. pneumoniae*. SCVs are characterized by their small size, slow growth, and altered metabolic activity, often accompanied by persistence in hostile conditions such as antibiotic treatment or host immune defenses (12, 13). *K. pneumoniae* isolates typically form dome-shaped, smooth, shiny, and white or whitish colonies on blood agar, regardless of their pathotype. While SCVs are commonly described in staphylococcal species (12, 14) and, to a lesser extent, in gram-negative bacteria such as *Escherichia coli* and *Pseudomonas aeruginosa* (15, 16), *K. pneumoniae* also displays other morphological variations under specific conditions, such as exposure to antibiotics (17) or phages (18). These variations include altered colony surface structures (18) and changes in single-cell morphology (7, 19). Importantly, these changes are linked to capsule modifications, impacting antibiotic susceptibility (19), biofilm formation (7), and virulence (18). For example, one of our previous studies described a *K. pneumoniae* isolate with sponge-like colonies on blood agar, which produced increased levels of biofilm-related cellulose and contained insertion sequences (ISs) within its K and O loci (20).

This study investigates the potential *in vivo* evolution and morphotypic diversity of the consecutive *K. pneumoniae* ST147 isolates, focusing on either the normal white colonies or the different morphotypes. Through genomic and phenotypic analyses—including measurements of exopolysaccharides and siderophores—we elucidate the adaptive evolution of cKp transitioning to convergent *K. pneumoniae* within the host. Additionally, we uncover genomic and transcriptomic adaptations that help to explain the development of the different morphotypes.

## MATERIALS AND METHODS

### Bacterial samples

The *K. pneumoniae* isolates used in this study are listed in Table 1. All isolates were stored at −80°C in lysogeny broth containing 20% (vol/vol) glycerol (anhydrous; Merck, Darmstadt, Germany). Unless otherwise stated, isolates were cultured at 37°C on Columbia blood agar plates supplemented with 5% sheep blood (Mast Diagnostica, Reinfeld, Germany). A reference strain of hvKp (PBIO2030; ST420) was used as a positive control for the siderophore secretion assay, uronic acid assay, and the *Galleria mellonella* infection model (21).

**TABLE 1:** Overview of *K. pneumoniae* isolates used in this study (based on reference 11)

Isolate ID	Sampling time point and date (mo-day-yr)	Origin/isolation site	Phenotypic characteristics
PBIO4684 (1A)	1 (07-19-2022)	Urine	White morphotype (stable)
PBIO4685 (1B)			g/d morphotype (stable)
PBIO4686 (2A)	2 (07-22-2022)	Intraoperatively obtained hip tissue	White morphotype (stable)
PBIO4687 (2B)			Gray morphotype (stable)
PBIO4688 (3A)	3 (08-01-2022)	Intraoperatively obtained hip tissue	White morphotype (stable)
PBIO4689 (3B)			Gray morphotype (stable)
PBIO4690 (4A)	4 (09-16-2022)	Intraoperatively obtained hip tissue	SCV (unstable)
PBIO4691 (4B)			White morphotype (stable)
PBIO4692 (4C)			Gray morphotype (stable)
PBIO4693 (4D)			g/d morphotype (stable)
PBIO4694 (5A)	5 (09-28-2022)	Intraoperatively obtained hip tissue	SCV (unstable)
PBIO4695 (5B)			White morphotype (stable)
PBIO4696 (5C)			Gray morphotype (stable)
PBIO4697 (5D)			g/d morphotype (stable)

### Genomic analyses

Short-read sequencing data for all 14 isolates (i.e., all morphotypes of isolates 1–5) were obtained in a previous study (11) and were deposited under BioProject PRJEB71325. In addition, in this new study, hybrid sequencing was performed for isolates 5A-D. DNA was extracted using the MasterPure DNA Purification kit for Blood, v.2 (Lucigen, Middleton, WI, USA) according to the manufacturer’s instructions and quantified with the Qubit 4 fluorometer and the dsDNA HS Assay kit (Thermo Fisher Scientific, Waltham, MA, USA), which suggested that DNA quantity and quality were sufficient for sequencing. Samples were sequenced at the Helmholtz Center for Infection Research (Braunschweig, Germany) using Illumina short-read technology (NovaSeq 6000, 2 × 150 bp reads) and Oxford Nanopore long-read technology (FLO-MN106D, R9.4.1, MinION platform) sequencing. Illumina short reads were assessed for quality with FastQC v.0.11.9 (https://www.bioinformatics.babraham.ac.uk/projects/fastqc/) and MultiQC v.1.14 (22) before and after quality and adapter trimming with fastp v.0.23.2 (23, 24). Hybrid assemblies were generated from long- and short-reads using Unicycler v.0.5.0 (25), and the resulting closed genomes were visualized using Bandage v.0.8.1 (26). Genome annotation was performed using bakta v.1.7.0 and its database v.5.0 (27). To identify potential mutations, short-read data from BioProject PRJEB71325 were analyzed with breseq v.0.38.1 (28), using the closed genome sequence of isolate 5B (white morphotype) as a reference. The results were manually inspected and compared across isolates. Antibiotic resistance and virulence detection were performed using ABRicate v.1.0.0 (https://github.com/tseemann/abricate, accessed on 16 January 2023; using ResFinder [29], PlasmidFinder [30], and VFDB [31] databases), and Kleborate v.2.2.0 (32) with Kaptive v.2.0.0 (33).

### Gene expression analysis

Gene expression of isolates 5A-D was analyzed by RNA sequencing and differential gene expression (DGE) analysis. The isolates were grown on blood agar plates and incubated overnight at 37°C. Total RNA was extracted using the RNeasy kit (Qiagen, Hilden, Germany) and purified using the rDNase and RNA Clean-up kit (Macherey-Nagel, Düren, Germany). RNA quality was assessed using the Prokaryote Total RNA Nano Series II Chip on the Agilent Bioanalyzer 2100 (Agilent Technologies, Santa Clara, CA, USA). RNA samples were sent to Novogene (Cambridge, UK) for sequencing using NovaSeq 6000 S4 platform (paired-end, 2 × 150 bp reads; stranded). Trimming of adapters and quality of RNA short reads was performed using Trim Galore v.0.6.8 (https://www.bioinformatics.babraham.ac.uk/projects/trim_galore/), followed by quality assessment using FastQC and MultiQC. The trimmed reads were mapped to the closed reference assembly of 5B using bowtie2 v.2.5.1 (34) and formatted with samtools v.1.13 (35). FeatureCounts v.2.0.1 (stranded mode) (36) was used to count read mappings to coding features. Differential gene expression analysis was performed in R v.4.3.1 (https://www.r-project.org/) with DESeq2 v.1.40.0 (37) with design formula: ~batch + condition. Genes were considered as differentially expressed if they had an absolute log2 fold change (log2FC) ≥2 and the adjusted *P* value < 0.05.

### Confirmation of chromosomal plasmid integration

Chromosomal plasmid integration was analyzed using custom primers (PP1 and PP2) and primers for the housekeeping gene *infB* (Eurofins GSC Lux, Luxembourg, Luxembourg). All oligonucleotide sequences are summarized in Table S1.

For each sample, DreamTaq Green PCR master mix (Thermo Fisher Scientific, Waltham, MA, USA), 0.5 µM of each primer, and 50 ng DNA template were used. PCR amplification was performed with the following conditions: initial denaturation at 95°C for 5 min; 30 cycles of denaturation at 95°C for 30 s, annealing at 58°C (PP1 and PP2) or 53°C (*infB*) for 30 s, and elongation at 72°C for 4 min 30 s (PP1 and PP2) or 1 min (for PP1_fw with PP2_rv and *infB*); followed by a final elongation at 72°C for 10 min. PCR products were separated on a 1.2% agarose gel stained with 0.01% (vol/vol) GelGreen Nucleic Acid Stain (Merck, Darmstadt, Germany) in Tris-Borate-EDTA buffer and run at 120 V for 2 h. GeneRuler 1 kb Plus DNA ladder (Thermo Fisher Scientific, Waltham, WA, USA) was used as a size marker. Gel images were documented with Quantum CX5 and BioVision v.17.06 software (Vilber, Collégien, France).

### Quantification of uronic acids

Uronic acid content, as a measure of capsule polysaccharides, was quantified using a modified carbazole assay as described previously (38). Briefly, multiple single colonies were resuspended in 1 mL of distilled water, and the OD600_600_ was determined. Capsule polysaccharides were extracted by incubating the suspension with phenol at 68°C for 30 min. After cooling to room temperature, chloroform was added, and the aqueous phase was separated by centrifugation at 12,000 × *g* for 10 min. The polysaccharides were precipitated by mixing the aqueous phase with ethanol and incubating the mixture at −20°C. The resulting precipitate was resuspended in 1 mL of distilled water. For the carbazole assay, the extracted polysaccharides were mixed with 25 mM sodium tetraborate dissolved in sulfuric acid and heated at 100°C for 10 min. After cooling to room temperature, an ethanolic carbazole solution was added to the mixture, which was then heated for an additional 15 min. Once the samples cooled to room temperature, OD530_530_ was measured. Uronic acid concentrations were determined by comparing the OD530_530_ readings to a standard curve generated using glucuronic acid and normalized to the OD600_600_ of the bacterial suspension.

### Siderophore secretion

The ability to produce siderophores was tested as described previously (39). Briefly, colonies were suspended in 0.9% (wt/vol) NaCl until OD600_600_ reached 0.5 McFarland standard turbidity and diluted 1:100 in 5 mL iron-chelated M9 medium (200 µM 2,2′dipyridyl [Carl Roth, Karlsruhe, Germany] added to M9 medium) supplemented with 0.3% (dipyridyl [Carl Roth, Karlsruhe, Germany] added to M9 medium) supplemented with 0.3% (wt/vol) casamino acids (BD, Franklin Lakes, NJ, USA) in sterile 15 mL tubes (Sarstedt, Nürnbrecht, Germany). Following overnight incubation at 37°C and 130 rpm on a rotary shaker, 1 mL of the suspension was placed in 1.5 mL tubes and centrifuged at 4,900 × *g* and room temperature (RT) for 20 min. In a 96-well plate, 100 µL of the supernatant was mixed with 100 µL chrome azurol S shuttle solution (prepared according to reference 39), 15 mM aqueous EDTA solution (Carl Roth, Karlsruhe, Germany) mixed with the shuttle solution was used as positive control, and the plate was incubated in the dark for 30 min at RT. The absorption was measured at *λ*  =  630 nm with the microplate reader, and siderophore production was calculated as previously described (40) and expressed as a percentage unit of siderophore production.

### *G. mellonella* infection model

Larvae of the greater wax moth *G. mellonella* were infected with the isolates to test mortality as described previously (41). Briefly, single colonies were suspended in phosphate buffered saline (PBS) until an OD600_600_ of 1 (approx. 2 × 10^9^ CFU/mL) was reached. After centrifugation at 12,000 × *g* and RT for 5 min, the bacterial suspensions were washed twice with PBS and diluted to 2 × 10^7^ CFU/mL. Then, larvae were randomly divided into groups of 10 individuals, and 10 µL of the adjusted suspension was injected into the left proleg. Additionally, 10 µL of PBS was injected into another group of larvae (*n* = 10) to exclude death due to injection trauma. Each group was placed into 90 mm petri dishes and kept in the dark at 37°C. Death was recorded every 24 h, and larvae were considered dead when they no longer responded to physical stimuli and showed pigmentation. Results for the replicates of each isolate were pooled, and Kaplan-Meier plots were used to show mortality rates (42). Although the *G. mellonella* model has certain limitations, such as differences in host immune responses compared to mammals, it remains a valuable tool for assessing bacterial virulence (43, 44). This model is particularly useful when combined with other assays, allowing for a more comprehensive understanding of bacterial pathogenicity.

### Statistical analyses

Statistical analyses were performed with GraphPad Prism v.7.05 (GraphPad software, San Diego, CA, USA). Phenotypic experiments were performed with at least three biological triplicates, with each biological replicate subdivided into technical triplicates. Unless otherwise stated, data are expressed as means ± SEM. Statistical significance was determined with an analysis of variation (ANOVA) with Dunnett’s *post hoc* test, comparing all isolates to 1A. Statistical significance was defined as a *P*-value < 0.05. Synteny plots were generated using pyGenomeViz (https://github.com/moshi4/pyGenomeViz), and images were created with BioRender.

## RESULTS

### Acquisition of plasmid-encoded resistance and virulence genes results in transition from classic to convergent *K. pneumoniae* within the host

**Fig 1:**
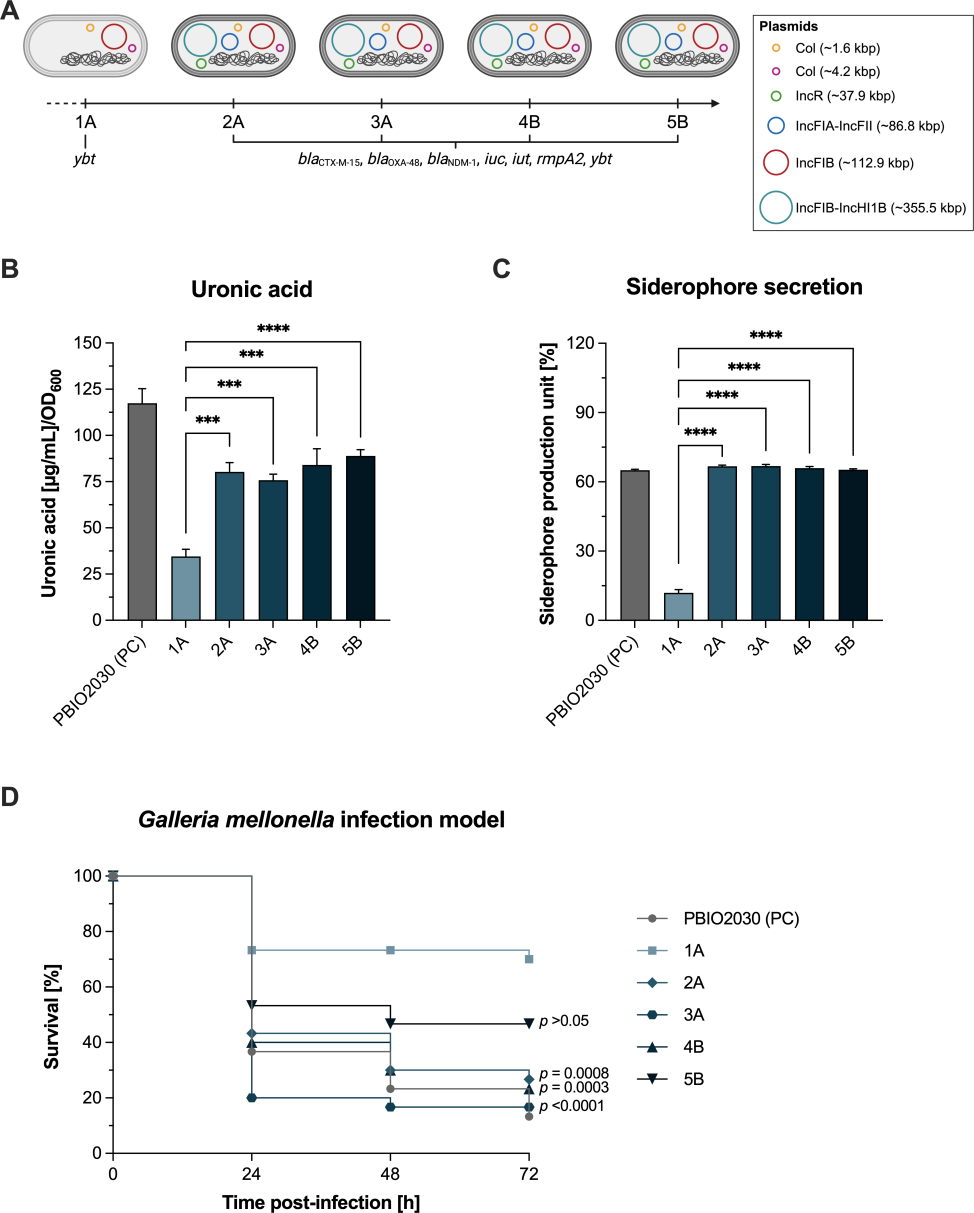
Temporal dynamics of plasmid carriage and virulence-associated features in white phenotype isolates. (**A**) Schematic representation of plasmid carriage in the isolates. Plasmid carriage in isolates 2A to 5B introduced additional virulence genes characteristic of hypervirulent *K. pneumoniae*, including the aerobactin translocation system (*iuc*, *iut*) and the mucoid phenotype regulator (*rmpA2*). Notably, the *rmpA2* gene was only partially present, with approximately 40% coverage. (**B**) Quantification of capsular polysaccharides by uronic acid assay (*n* = 3). Data are expressed as mean ± standard error of the mean. (**C**) Siderophore secretion levels expressed as mean percentage units of siderophore production ± standard error of the mean (*n* = 3). (**D**) Kaplan-Meier survival analysis of *G. mellonella* larvae after infection (*n* = 30 larvae/condition). Results are expressed as mean percent mortality after injection of 10^5^ CFU per larva. Statistical comparisons for uronic acid and siderophore secretion assays were conducted using one-way ANOVA with Dunnett’s post hoc test against isolate 1A, and for the survival analysis, log-rank tests were performed against isolate 1A; significance levels are indicated as ***, *P* < 0.001; ****, *P* < 0.0001. PC: positive control.

Since not all morphotypes were stable, the evolutionary analysis focuses on the “normal” isolates, while the non-normal morphotypes are addressed separately, with a discussion on the underlying mechanisms. According to the findings of Doğan et al., all *K. pneumoniae* isolates were identified as belonging to ST147 and to the same clonal lineage, as evidenced by the low number of single-nucleotide polymorphisms (SNPs) between them (11). Plasmid analysis revealed different incompatibility group (Inc) profiles across the isolates (Fig. 1A; Table S2). The first isolate with the white morphotype (1A) carried only two Col plasmids (~1.6 kbp and ~4.2 kbp) and one IncFIB plasmid (~112.9 kbp), whereas the following isolates with the white phenotype (i.e., 2A, 3A, 4B, and 5B) additionally carried an IncR (~37.9 kbp) and an IncFIB plasmid (~112.9 kbp) as well as two multireplicon plasmids (IncFIA-IncFII, ~86.8 kbp; IncFIB-IncHI1B, ~355.5 kbp). The plasmid acquisition significantly influenced the antimicrobial resistance (AMR) and virulence-associated gene content of the isolates. Isolate 1A lacked the IncFIB-IncHI1B plasmid, which carried important AMR features, including the extended-spectrum β-lactamase (ESBL) gene *bla*_CTX-M-15_ and the carbapenemase genes *bla*_OXA-48_ and *bla*_NDM-1_. As a result, resistance scores by Kleborate criteria were lowest in 1A (0 vs 2 in the later isolates). The virulence score was similarly low (1 vs 4 in later isolates), as isolate 1A only harbored yersiniabactin (*ybt*), while the other isolates were also positive for aerobactin (*iuc*, *iut*). In addition, the *rmpA2* gene (regulator of mucoid phenotype), a biomarker for hvKp, was absent in isolate 1A. Note that the *rmpA2* gene itself was only partially present in the isolates in which it was detected, with a coverage of about 40%.

### Phenotypic results confirm increased virulence in convergent isolates

To investigate the microevolution from classic to the convergent *K. pneumoniae* pathotype on phenotypic levels, we further analyzed the five isolates with the white morphotype and included a positive hvKp control (PBIO2030). The assessment of phenotypic virulence included tests to determine the expression of capsular polysaccharides (uronic acid assay), siderophore secretion, and an *in vivo* infection model using *G. mellonella* larvae. Capsular polysaccharide production (Fig. 1B) was lowest in isolate 1A (*P* < 0.0001) compared to all subsequent isolates (2A, 3A, 4B, and 5B), which displayed similar levels of capsule production. Secretion of siderophores (Fig. 1C), a critical virulence factor, was also significantly lower in 1A (*P* < 0.0001) compared to the other isolates, which had similar levels as the positive control. Finally, in the *G. mellonella* infection model (Fig. 1D), isolate 1A showed the highest survival rates among the five isolates, with nearly 70% of larvae surviving after 72 h, indicating the lowest virulence. Survival decreased in the other isolates, with isolate 3A exhibiting the highest mortality, similar to the positive control.

### Detection of chromosomal integration of a hybrid plasmid suggests stable fixation of beneficial traits in one isolate

**Fig 2:**
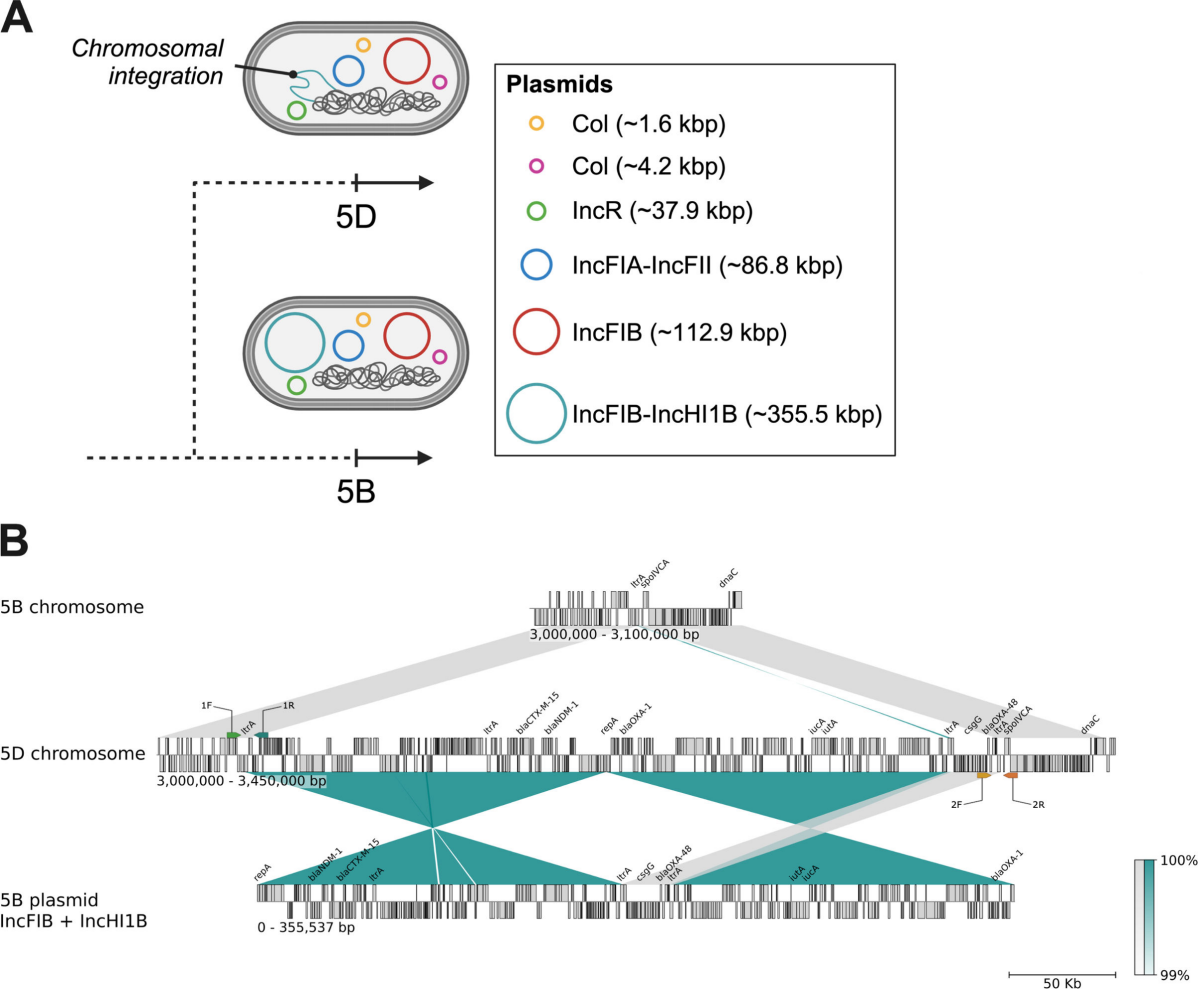
Chromosomal integration of a multireplicon IncFIB-IncHI1B plasmid in isolate 5D. (**A**) Schematic representation of the plasmid content and chromosomal integration event in isolate 5D compared to isolate 5B. (**B**) Synteny plot comparing the chromosome of isolate 5B, the chromosome of isolate 5D, and the IncFIB-IncHI1B plasmid. The alignment shows the integration of the IncFIB-IncHI1B plasmid region (highlighted in teal) into the 5D chromosome. The position of the custom primer pairs used to demonstrate chromosomal plasmid integration is indicated by arrows above (1F and 1R) and below (2F and 2R) the chromosome of 5D. Selected resistance and virulence genes, such as *bla*_CTX-M-15_, *bla*_NDM-1_, *iutA*, and *iucA*, are annotated for clarity. The corresponding regions have high sequence similarity (99%–100%), as indicated by the gradient scale, and show the extensive genomic rearrangements associated with plasmid integration. For simplicity, the *iucBCD* operon has been omitted from the annotations.

Detailed analysis of the closed genome of isolate 5D revealed that the hybrid IncFIB-IncHI1B plasmid was integrated into the bacterial chromosome (Fig. 2A). This integration occurred between the genes *ltrA* (group II intron reverse transcriptase/maturase) and *spoIVCA* (DNA recombinase/invertase; Fig. 2B). Chromosomal integrations of large plasmids are often facilitated by group II introns through reverse splicing that allows direct insertion of genomic information into the chromosome (45). Compared with the closed plasmid sequence of the 5B reference, the integrated region in 5D retained most of the plasmid content but showed specific alterations: a smaller region, including *bla*_OXA-48_ and *csgG* (involved in biofilm formation), was preserved in the same orientation, while two larger regions containing the ESBL and carbapenemase genes, as well as the aerobactin operon, were inverted (Fig. 2B). PCR analysis (Fig. S1) confirmed that these “plasmid-based” genes were exclusively present in the chromosome of 5D, while absent in the chromosomes of 5A-C.

### Variations in the genomic K locus and adapted regulation of capsular genes likely contribute to the different morphotypes

**Fig 3:**
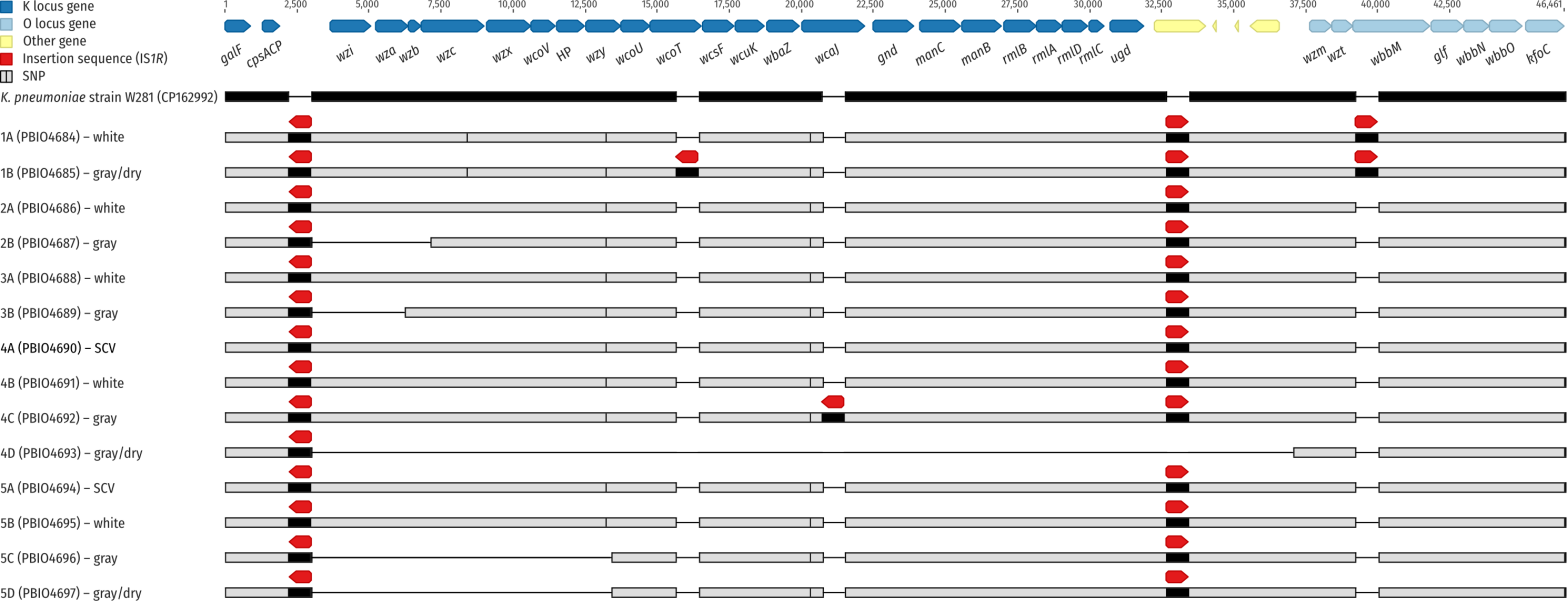
Overview of K and O locus interruptions and deletions. The multiple sequence alignment shows the K (KL64) and O (O1/O2v1) loci of all isolates in comparison to the public reference sequence of *K. pneumoniae* strain W281 (CP162992). Filled black rectangles in the reference show the reference backbone, while horizontal black lines indicate gaps. Gray rectangles for the isolates indicate sequences identical with the reference, while horizontal black lines indicate gaps, and vertical lines indicate SNP positions (see legend). Black rectangles for the isolates indicate insertions (IS*1R*).

**Fig 4:**
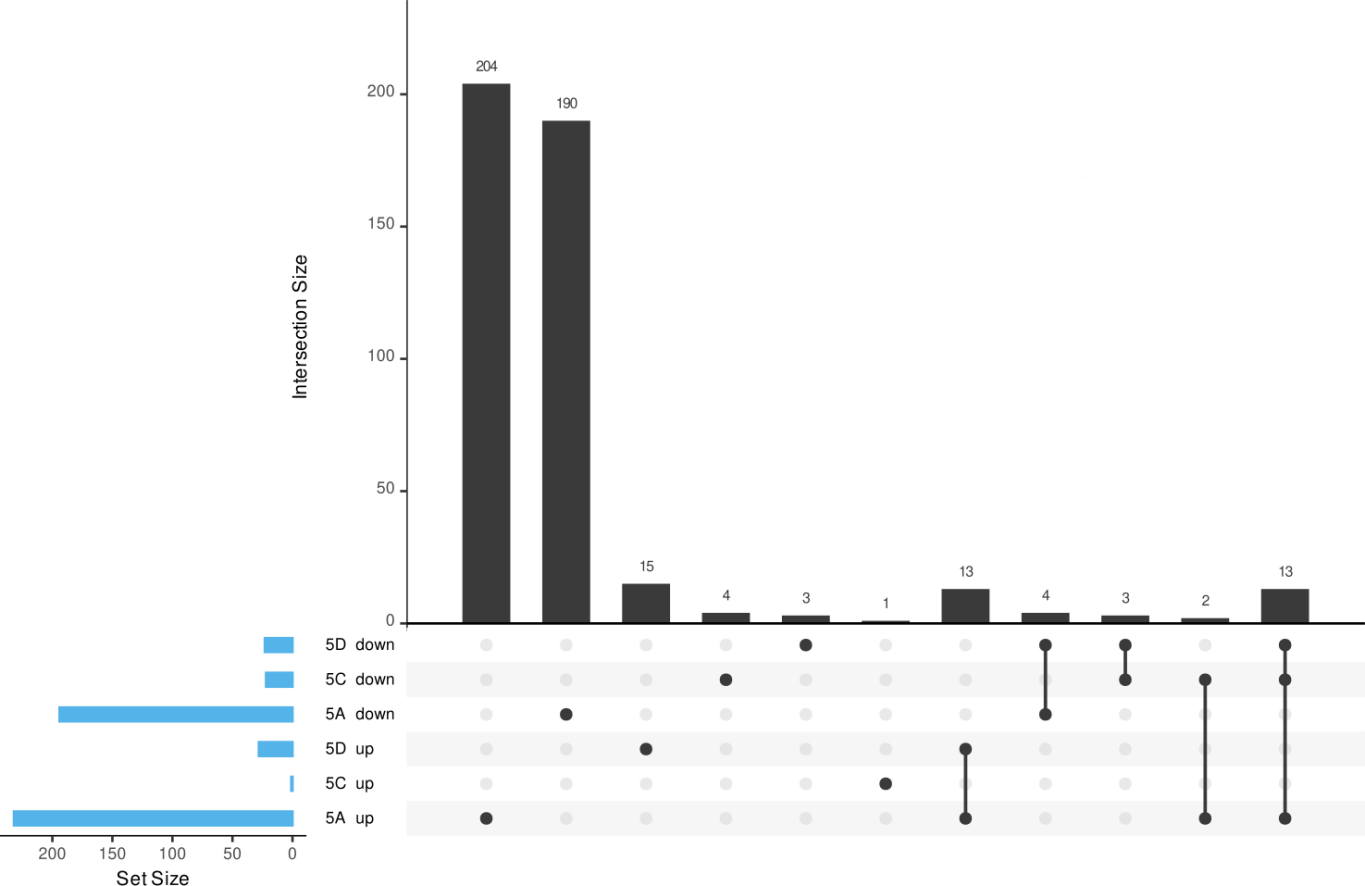
UpSet plot representing shared and uniquely DEGs in isolates 5A-D. All phenotypes (5A: small; 5C: gray; 5D: g/d) were compared to the white morphotype (5B), and only significantly different genes are shown (log2FC ≥ 2). Bars indicate the number of significantly up- or downregulated genes per isolate. Overlapping responses (intersections) are defined below the bars with connected dots. As the UpSet plot breaks down the DEGs into overlaps between isolates, they need to be summed accordingly to obtain the full count per isolate.

All isolates had an O locus of O1/O2v1 and a KL64 capsule biosynthesis (K) locus, except for isolate 4D, which lacked most of its K locus (11). Notably, isolates displaying gray or g/d phenotypes lacked at least one K locus gene (Fig. 3; Table S2). All isolates carried the insertion sequence IS*1R* (IS*1* family, 768 bp) within the K locus, and, except for 4D, this sequence was also found between the K and O loci, disrupting an acyltransferase gene. Additionally, IS*1R* was identified within the O locus (inserted into *wbbM*) in isolates 1A and 1B. Regarding the K locus, isolates 1A and 1B exhibited a missense mutation in *wzc* compared to the closed reference 5B (white; c.1615C > A; p.P539T). All isolates, except for 4D, 5C, and 5D, carried a silent mutation in *wzy* (c.717A > G; p.V239V), and all isolates except 4D carried an additional missense mutation in *wcaJ* (c.305_306CG > AA; p.T102K). In isolates 2B and 3B, deletions within the K locus, mediated by IS*1R*, affected the genes *wzi*, *wza*, *wzb*, and *wzc* in 2B, and *wzi* and *wza* in 3B, respectively. Further IS*1R*-mediated large deletions nearly eliminated the entire K locus in isolate 4D, leaving only *galF* and *cpsACP* intact, and removed genes *wzi*, *wza*, *wzb*, *wzc*, *wzx*, *wcoV*, a hypothetical protein-coding gene, and *wzy* in isolates 5C and 5D. Additionally, IS*1R* interrupted single K locus genes *wcoT* in isolate 1B and *wcaJ* in isolate 4C. We performed additional virulence assays for isolates 5B-D (Fig. S2). Isolate 5A was excluded due to the instability of the SCV, as overnight cultures could not reliably ensure testing of a consistent morphotype. Uronic acid assays indicated significantly lower exopolysaccharide production in 5C and 5D, aligning with the genomic disruptions observed in the K locus. Biofilm formation experiments revealed that biofilm production in 5C was comparable to 5B but significantly decreased in 5D. Mortality in the *G. mellonella* infection model was particularly high in 5C and 5D isolates; however, it was only statistically different for 5D. Then, transcriptome profiling was conducted on a selected group of isolates (5A-D). The decision to perform RNA sequencing on these isolates was primarily due to their specific makeup. Set 5 includes all four different morphotypes, including one strain with a chromosomally inserted hybrid plasmid. For the DGE analysis, isolate 5B, which exhibited the white morphotype, was used as the reference. The analysis revealed substantial variation in the number of differentially expressed genes (DEGs) across the morphotypes. Isolates 5C and 5D displayed 23 and 52 DEGs, respectively, which were considerably fewer than the 426 DEGs identified in isolate 5A (Fig. 4; Table S3). These variations were further underscored by principal component analysis, where isolates 5B, 5C, and 5D clustered closely together with only minor differences along PC1 when compared to 5A (Fig. S3). In contrast, isolate 5A separated distinctly along PC1, indicating its unique transcriptomic profile. All three isolates showed DEGs within the K locus compared to 5B. In isolates 5C and 5D, the absence of K locus genes led to a significant downregulation of nearly all genes involved in capsule biosynthesis (Table 2). In isolate 5A, however, most of these capsule genes were significantly upregulated.

**TABLE 2:** Overview of K locus genes that were significantly differentially expressed compared to isolate 5B^*b*^

Locus ID	Gene name	5A	5C	5D
PB4695KP_08500^*a*^	*wzi*	n.s.	−11.9	−13.06
PB4695KP_08505^*a*^	*wza*	n.s.	−11.56	−11.76
PB4695KP_08510^*a*^	*wzc*	2.46	−11.65	−11.86
PB4695KP_08515^*a*^	*wzx*	2.33	−9.34	−9.55
PB4695KP_08520^*a*^	*wcoV*	2.31	−9.28	−9.49
PB4695KP_08525^*a*^	hypothetical protein	2.46	−10.41	−10.62
PB4695KP_08530^*a*^	*wzy*	n.s.	−9.82	−6
PB4695KP_08535	*wcoU*	2.08	−4.62	−3.86
PB4695KP_08540	*wcoT*	2.59	−4.86	−6.01
PB4695KP_08545	*wcsF*	3.01	−8.98	−6.69
PB4695KP_08550	*wcuK*	2.98	−5.66	−5.17
PB4695KP_08555	*wbaZ*	3.24	−4.27	−3.31
PB4695KP_08560	*wcaJ*	3.12	−5.04	−4.64
PB4695KP_08570	*manC*	2.97	n.s.	n.s.
PB4695KP_08575	*manB*	2.71	n.s.	n.s.
PB4695KP_08580	*rmlB*	3.03	−2.4	−2.11
PB4695KP_08585	*rmlA*	2.76	−2.39	n.s.
PB4695KP_08590	*rmlD*	2.7	−2.23	−2.16
PB4695KP_08595	*rmlC*	2.71	n.s.	n.s.
PB4695KP_08600	*ugd*	2.7	−2.26	n.s.

^*a*^ Genes missing in isolates 5C and 5D; n.s.: non-significant, *P*-value (≥0.05) and/or log2FC (<2).

^*b*^ Only genes with significant differences in at least one isolate were included. Positive values indicate upregulation, while negative values correspond to downregulation of the respective gene, relative to the normal phenotype of 5B.

**Fig 5:**
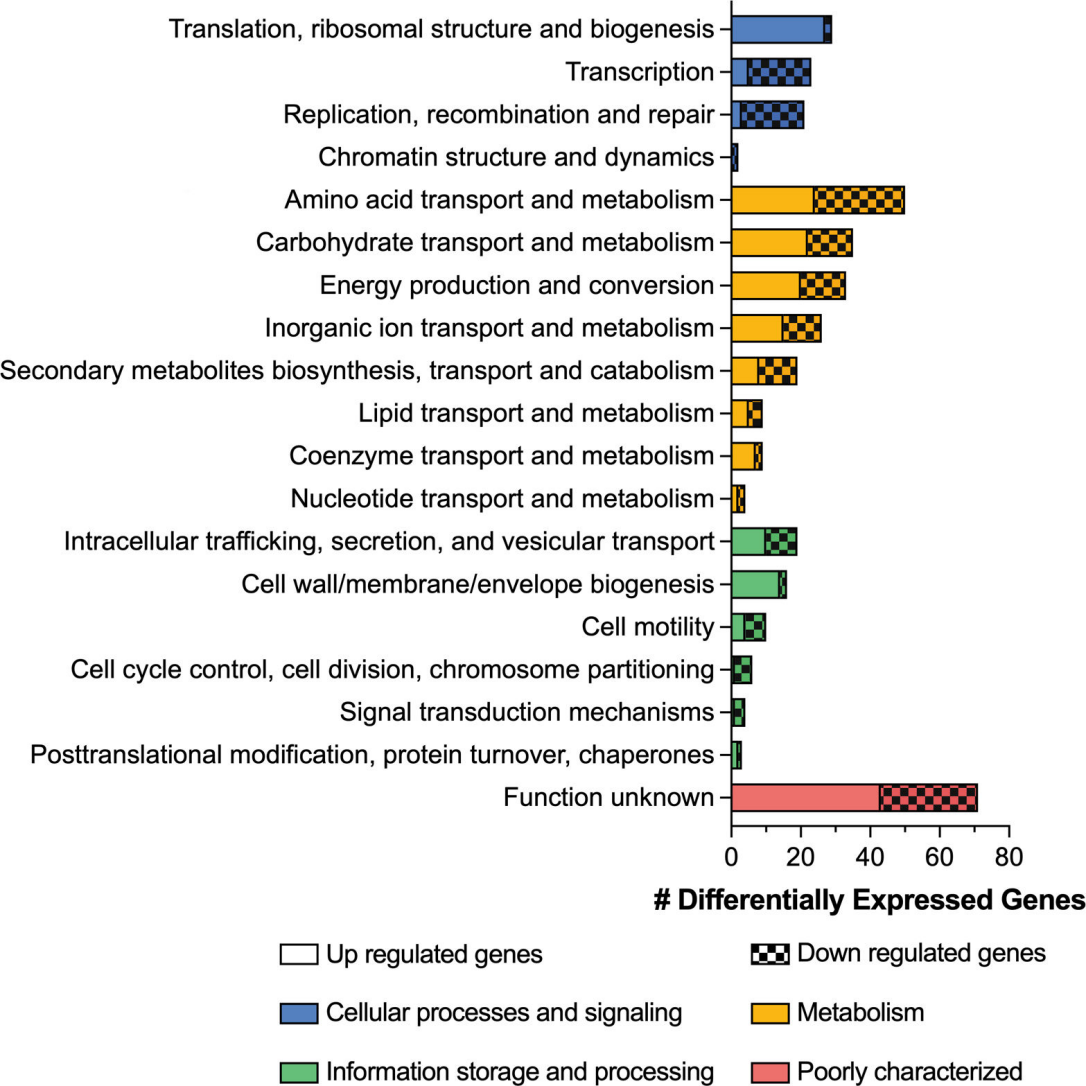
COG analysis for DEG in isolate 5A.

To further elucidate the phenotype-associated mechanisms, a cluster of orthologous groups (COG) analysis of the DEGs in isolates 5A, 5C, and 5D was performed. COG annotation revealed that only ~50% of genes in isolates 5C and 5D could be assigned to COG categories, with the most abundant category being cell wall/membrane/envelope biogenesis (M), corresponding to downregulated genes located in the K locus (Table S4). The main regulated categories for isolate 5A included energy production and conversion (C), amino acid transport and metabolism (E), and carbohydrate transport and metabolism (G). In addition, genes related to translation, ribosomal structure and biogenesis (J) and cell wall/membrane/envelope biogenesis (M) were predominantly upregulated, while genes related to transcription (K), replication, recombination and repair (L), and signal transduction mechanisms (T) were predominantly downregulated (Fig. 5). These results were confirmed by analyzing the Kyoto encyclopedia of genes and genomes (KEGG) pathways, which also showed upregulated genes involved in genetic processing information, e.g., ribosomal proteins, and downregulated genes, for example, ABC transporters (Table S5).

## DISCUSSION

The ability of *K. pneumoniae* to adapt through genomic and transcriptomic changes, particularly in high-risk clonal lineages such as ST147 (32, 46), contributes to its persistence as an MDR pathogen in clinical settings. In this study, we suggest that in-host evolution is marked by the acquisition of genomic traits and the emergence of diverse morphotypes, which likely reflect bacterial adaptations to selective pressures. However, it is important to consider that different sampling sites could represent separate colonization events. Despite this possibility, the genomic data strongly support a shared evolutionary trajectory among the isolates, reinforcing the concept of in-host evolution. The transition observed from isolate 1A, with low virulence and resistance scores, to later isolates exhibiting higher scores and associated phenotypes further supports in-host evolution toward a convergent pathotype. This transition was closely linked to the acquisition of a large IncFIB-IncHI1B hybrid plasmid. The acquisition of such hybrid plasmids, which carry both AMR and virulence genes, is increasingly recognized as a driver of *K. pneumoniae* convergence (47, 48). Notably, isolate 5D displayed chromosomal integration of this plasmid, an event facilitated by group II introns. Chromosomal integration likely stabilizes beneficial traits and reduces fitness costs associated with plasmid carriage (10, 21). While we acknowledge the limitation that routine diagnostic isolates do not allow for exhaustive characterization of all individual strains, making it theoretically possible that convergent types were present earlier and overlooked, this scenario is unlikely. We acknowledge that routine diagnostic isolates may not fully capture diversity within a bacterial population. It is therefore possible that resistance or virulence traits were already present at low levels in early subpopulations but were not detected using standard methods. These subpopulations may have been selected under antimicrobial or host pressure, leading to their expansion in later isolates.

Our investigation of the gray and g/d morphotypes revealed extensive deletions within the K locus, primarily mediated by IS elements. IS*1R* replication causes a target site duplication (direct repeat) of 8−9 bp upon insertion of the IS element, i.e., the element is flanked by the same short sequence. In the case of a deletion event between two nearby IS elements—leaving behind a single IS element—the left and right flanking sequences differ from each other as they originate from two different original insertion events. These deletions impaired capsule biosynthesis, resulting in reduced capsule production. Uronic acid assays further confirmed significantly lower exopolysaccharide production in 5C and 5D, aligning with the genomic disruptions in the K locus. Despite reduced capsule production, biofilm formation experiments revealed notable differences: 5C exhibited increased biofilm formation, whereas biofilm production remained comparatively low in 5D. The reduced biofilm formation in 5D may be linked to disruptions involving *csgG*, a key gene for the production of biofilm components (49). In isolate 5D, the *csgG*-containing region was preserved within the chromosomally integrated IncFIB-IncHI1B plasmid, yet structural rearrangements may have disrupted its regulatory or functional role, potentially impairing and reducing biofilm production. In contrast, isolate 5C, which does not carry such disruptions, displayed increased biofilm production, suggesting functional assembly of biofilm components as a contributing factor to the observed phenotype. Capsule production and biofilm formation are intricately linked but can also have opposing effects. While a thick capsule provides protection and facilitates surface adhesion, it can inhibit biofilm formation by masking adhesins critical for biofilm matrix development (50). The reduced exopolysaccharide production observed in 5C and 5D may therefore expose biofilm-promoting components. These findings align with previous studies showing that capsule-deficient *K. pneumoniae* strains often exhibit increased biofilm formation due to reduced capsule interference with surface adhesion (50, 51). Ernst et al. reported *K. pneumoniae* ST258 isolates that formed gray, flat colonies resembling our g/d morphotypes (7). These isolates were associated with large deletions and IS elements within the K locus, resulting in capsule deficiencies and primarily linked to urinary tract infections. This suggests that capsule loss does not necessarily limit pathogenic potential, as also supported by the high mortality observed in *G. mellonella* for isolates 5C and 5D. Notably, both isolates had the same Kleborate resistance and virulence scores as 5B, further emphasizing the clinical relevance of such morphotypes.

In contrast, the SCV phenotype observed in isolate 5A exemplifies a distinct survival strategy among *K. pneumoniae* isolates, driven primarily by transcriptional reprogramming. Unlike the capsule-deficient gray morphotypes, where extensive deletions in the K locus preclude capsule production, SCV formation in 5A was associated with significant upregulation of capsule and O-antigen biosynthesis genes. This suggests that increased capsule production may enhance persistence by providing additional protection against environmental stressors, balancing the hallmark SCV traits of slow growth and altered metabolism (12, 13). SCVs are a well-recognized adaptive strategy among bacterial pathogens, including *K. pneumoniae*, enabling survival under hostile conditions such as antibiotic treatment and immune responses. Characterized by their small size, slow growth, and metabolic shifts, SCVs can persist in a dormant-like state under stress and later re-emerge when conditions improve (12). While SCVs have been predominantly studied in *Staphylococcus aureus*, where they are associated with chronic infections and linked to persister cell subpopulations (12, 13), they are less frequently reported in gram-negative bacteria such as *E. coli* and *P. aeruginosa* (15, 16). In our study, transcriptomic analysis of SCV isolate 5A revealed substantial changes compared to its white morphotype counterpart (5B), with over 1,200 DEGs. Additionally, upregulated pathways included those related to energy production, iron acquisition, and carbohydrate metabolism, which may provide a survival advantage under nutrient-limited or stressful conditions. Conversely, genes involved in propanediol metabolism, glycerol transport, and cellulose biosynthesis were downregulated, reflecting metabolic shifts that likely contribute to the SCV’s reduced growth rate and altered phenotype. Interestingly, the SCV phenotype also showed significant upregulation of AMR genes such as *bla*_CTX-M-15_ and *bla*_OXA-48_, supporting a link between resistance gene expression and slower growth rates, as previously described in *E. coli* (52). Moreover, genes associated with persister cell formation, including *nuoI*, *glpD*, and *tisB*, displayed differential regulation. While some of these genes exhibited expression patterns consistent with bacterial persistence, others showed inverse trends, suggesting a complex and species-specific regulatory interplay in SCV development. It is important to note that we did not test the pathogenic potential of the SCVs directly, as their phenotypic instability precluded such assays. Due to the unstable nature of these morphotypes, overnight cultures—a prerequisite for many phenotypic assays—could not be reliably obtained. Consequently, while our analysis provides genomic and transcriptomic insights, we focused phenotypic characterization exclusively on the other morphotypes. This limitation underscores a challenge in studying such morphotypic variants: their instability hampers comprehensive functional characterization, including pathogenicity, persistence, and resistance phenotyping (53).

In conclusion, our study suggests that *K. pneumoniae* has the capacity to adapt and survive under selective pressures, as evidenced by genomic and phenotypic changes. The emergence of hybrid plasmid-driven convergent pathotypes, along with distinct survival strategies such as SCVs and capsule-deficient morphotypes, highlights the potential role of transcriptional reprogramming and structural changes in enhancing persistence in clinical environments.

## Data Availability

The data for this study have been deposited in the European Nucleotide Archive (ENA) at EMBL-EBI under the accession number PRJEB71325.

## SUPPLEMENTAL MATERIAL

Figures S1 to S3 spectrum.00170-25-s0001.docx Figure S1: Representative gel electrophoresis image of PCR products supporting the chromosomal plasmid integration of isolate 5D. Figure S2: Virulence-associated features in gray and g/d morphotype (5C and 5D, respectively) isolates compared to the white morphotype (5B). Figure S3: Principal component analysis (PCA) of isolates 5A-D.

Tables S1 to S5 spectrum.00170-25-s0002.xlsx Table S1: Summary of oligonucleotide sequences used in this study. Table S2: Results of whole-genome sequencing concerning virulence and resistance scores, K loci, and plasmids. Table S3: Differentially expressed genes compared to isolate 5B. Table S4: Cluster of orthologous groups (COG) analysis of differentially expressed genes. Table S5: KEGG categories of differentially expressed genes compared to isolate 5B.

ASM does not own the copyrights to Supplemental Material that may be linked to, or accessed through, an article. The authors have granted ASM a non-exclusive, world-wide license to publish the Supplemental Material files. Please contact the corresponding author directly for reuse.
