# Supplementary material for: Potential in-host evolution of Klebsiella pneumoniae ST147: convergence and the role of capsular alterations in morphotype diversity
Source: Microbiol Spectr. 2025 Jul 18;13(9):e00170-25. doi: 10.1128/spectrum.00170-25 (PMC12403872; doi:10.1128/spectrum.00170-25)

**Supplementary Figure S1:** Representative gel electrophoresis image of PCR products supporting the chromosomal plasmid integration of isolate 5D. Specific primer pairs were used for the amplification of the left and right junction sequences of the chromosome and integrated plasmid. The expected amplicons were sized 4,499 bp (primer 1F + 1R, left junction) and 4,882 bp (2F + 2R, right junction), respectively. Additionally, the primers 1F and 2R were combined to exclude that no insertion was present in isolate 5D and confirm there was no insertion for the other isolates (5A-C). Genomic DNA was used as template and the negative controls (NC) were substituted with water instead.


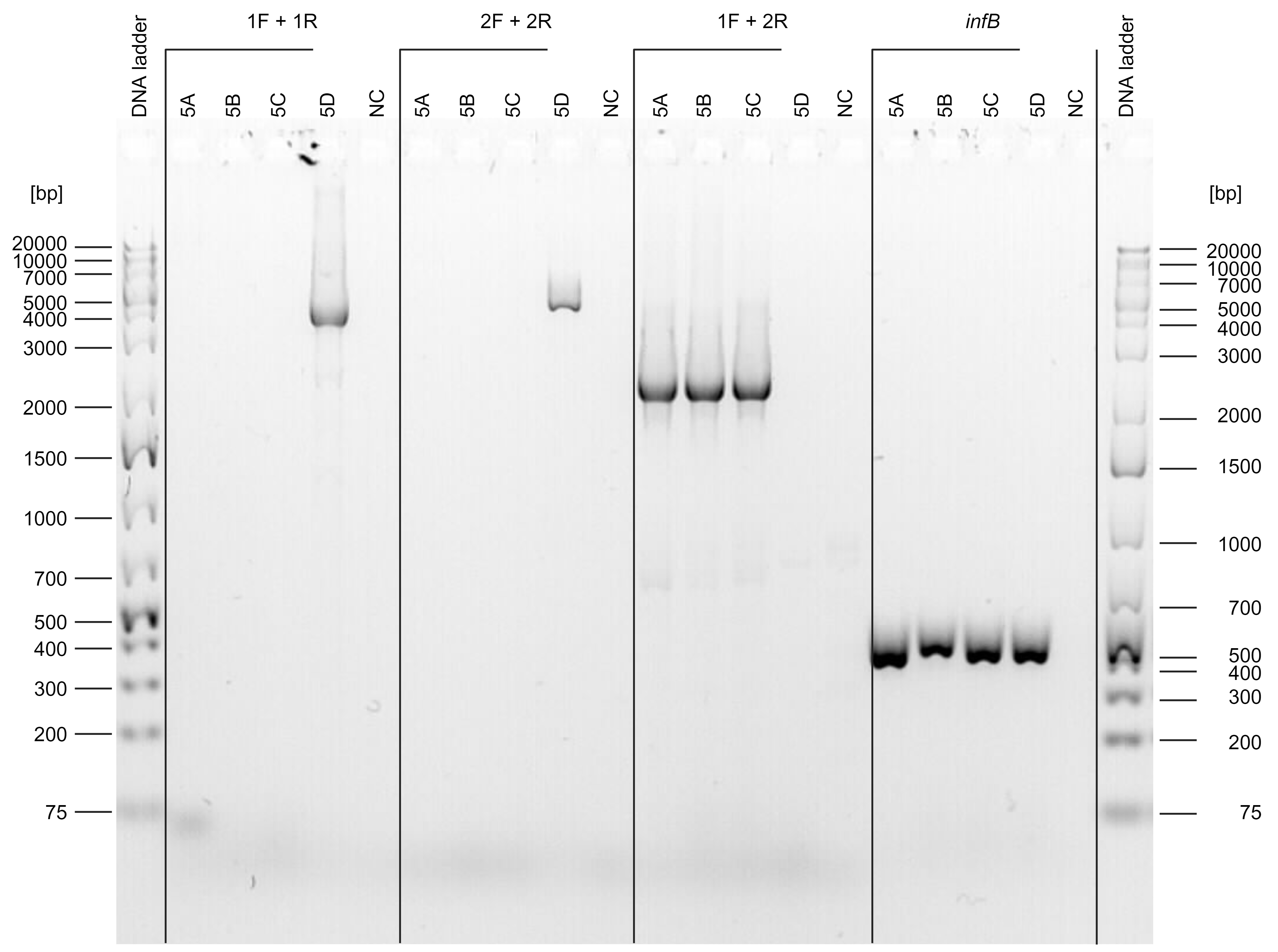


**Supplementary Figure S2:** Virulence-associated features in gray and g/d morphotype (5C and 5D, respectively) isolates compared to the white morphotype (5B). **A** Quantification of capsular polysaccharides by uronic acid assay (n = 3). Data are expressed as mean ± standard error of the mean. **B** Siderophore secretion levels expressed as mean percentage units of siderophore production ± standard error of the mean (n = 3). **C** Kaplan-Meier survival analysis of *Galleria mellonella* larvae after infection (n = 30 larvae/condition). Results are expressed as mean percent mortality after injection of 10^5^ CFU per larva. Statistical comparisons for uronic acid and siderophore secretion assays were conducted using one-way ANOVA with Dunnett's post hoc test against isolate 5B, and for the survival analysis, log-rank tests were performed against isolate 5B; significance levels are indicated as ***, *p* <0.001; ****, *p* <0.0001. PC: Positive control.


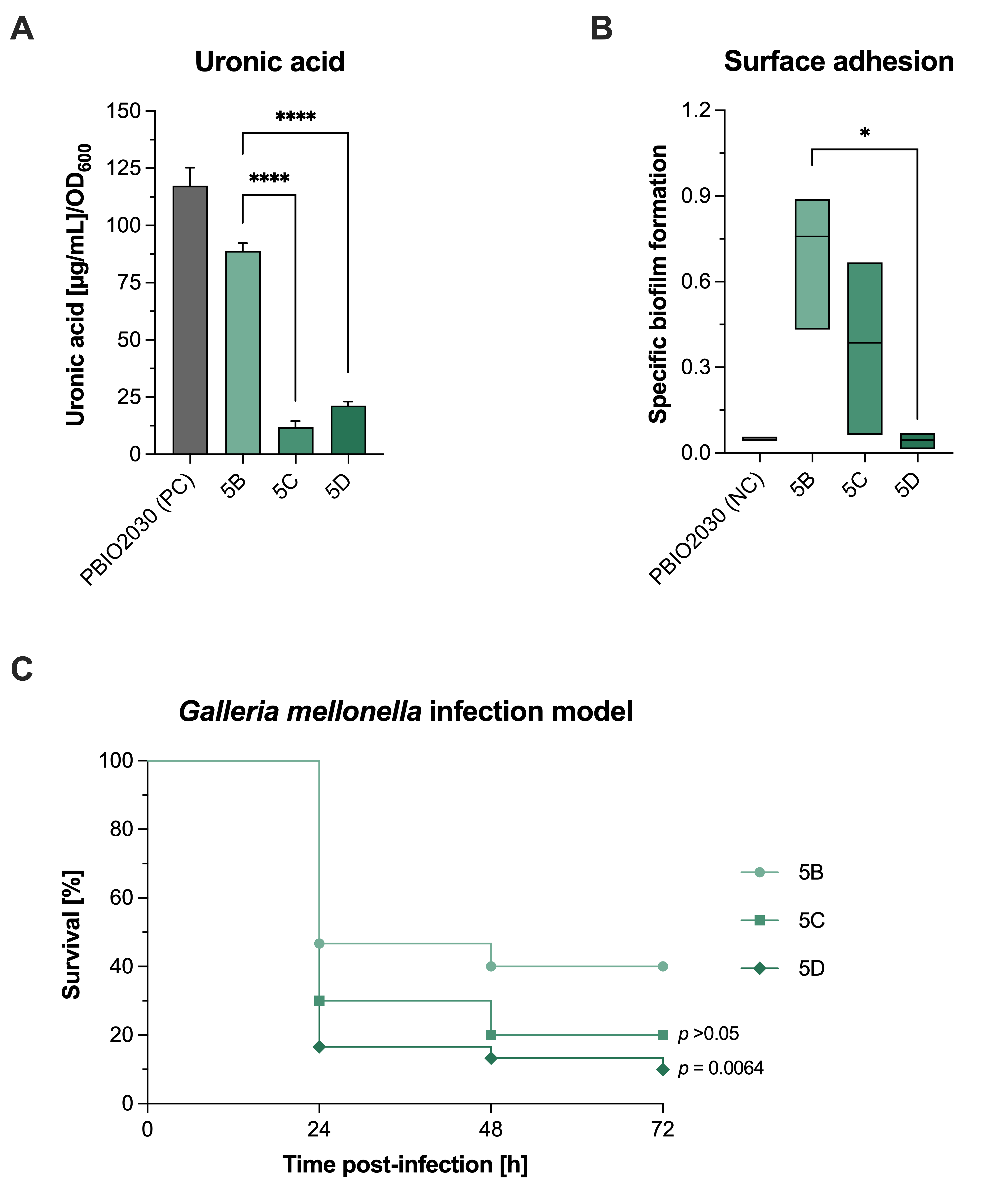


**Supplementary Figure S3:** Principal component analysis (PCA) of isolates 5A-D. PCA highlighted few variations in differentially expressed genes among the gray and g/d morphotypes (5C and 5D, respectively) compared to the white morphotype (5B), while indicating a unique transcriptomic profile for the small colony variant (SCV) phenotype (5A).
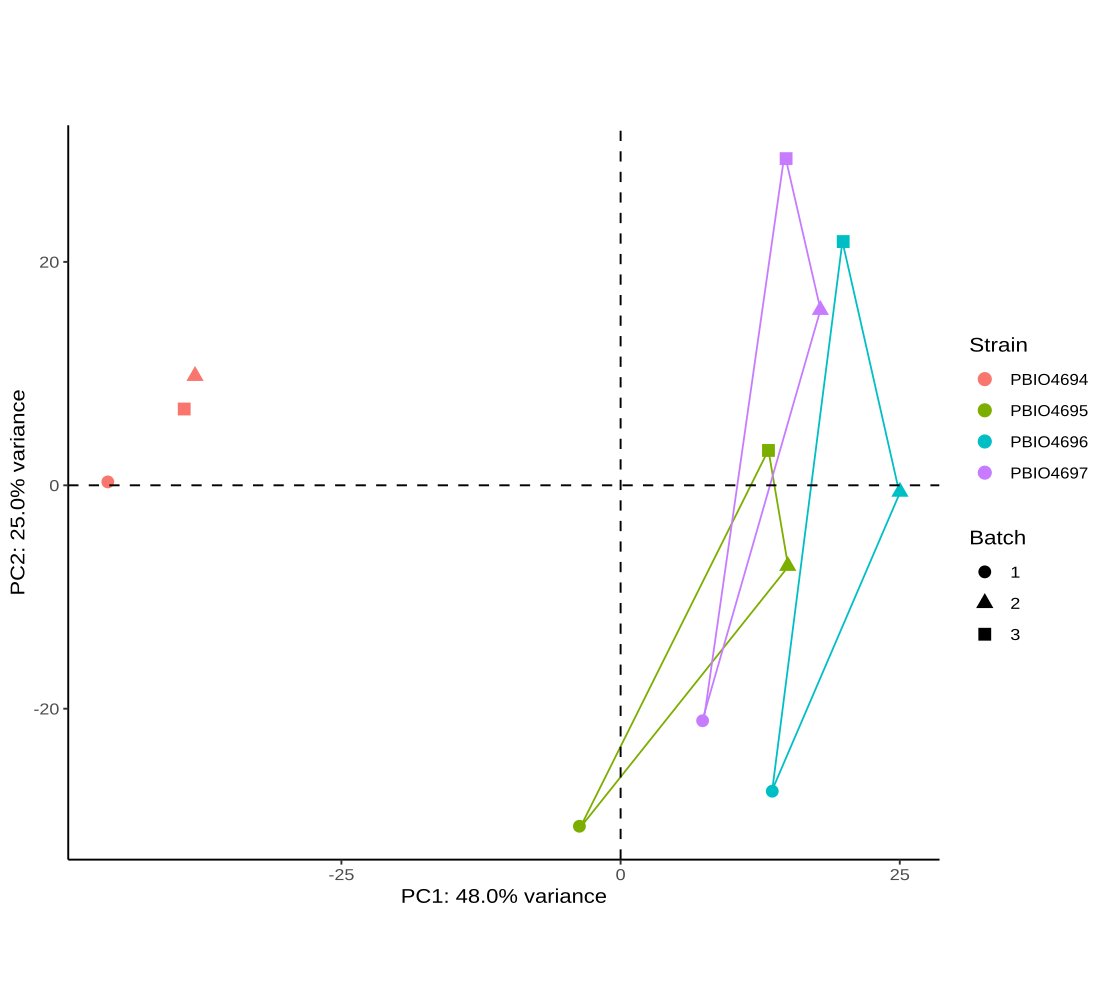

Supplement: Figures S1 to S3 — Figure S1: Representative gel electrophoresis image of PCR products supporting the chromosomal plasmid integration of isolate 5D. Figure S2: Virulence-associated features in gray and g/d morphotype (5C and 5D, respectively) isolates compared to the white morphotype (5B). Figure S3: Principal component analysis (PCA) of isolates 5A-D. [file spectrum.00170-25-s0001.docx]
